# Bladder cancer risk stratification with the Oncuria 10-plex bead-based urinalysis assay using three different Luminex xMAP instrumentation platforms

**DOI:** 10.1186/s12967-023-04811-2

**Published:** 2024-01-02

**Authors:** Hideki Furuya, Toru Sakatani, Sunao Tanaka, Kaoru Murakami, Richard T. Waldron, Wayne Hogrefe, Charles J. Rosser

**Affiliations:** 1 Cedars‑Sinai Medical Center, Samuel Oschin Comprehensive Cancer Institute, 110 N. George Burns Rd, Davis 2025, Los Angeles, CA 90048 USA; 2 Department of Biomedical Sciences, Cedars‑Sinai Medical Center, Los Angeles, CA USA; 3 Department of Urology, Cedars‑Sinai Medical Center, Los Angeles, CA USA; 4 Department of Medicine, Cedars‑Sinai Medical Center, Los Angeles, CA USA; 5 Nonagen Bioscience Corp., Los Angeles, CA USA

**Keywords:** Bladder cancer, Fluorescence, Multiplex immunoassay, Magnetic bead, In vitro assay, Performance, Dynamic range, Flow cytometry, xMAP technology

## Abstract

**Background:**

No single marker of bladder cancer (BC) exists in urine samples with sufficient accuracy for disease diagnosis and treatment monitoring. The multiplex Oncuria BC assay noninvasively quantifies the concentration of 10 protein analytes in voided urine samples to quickly generate a unique molecular profile with proven BC diagnostic and treatment-tracking utility. Test adoption by diagnostic and research laboratories mandates reliably reproducible assay performance across a variety of instrumentation platforms used in different laboratories.

**Methods:**

We compared the performance of the clinically validated Oncuria BC multiplex immunoassay when data output was generated on three different analyzer systems. Voided urine samples from 36 subjects (18 with BC and 18 Controls) were reacted with Oncuria test reagents in three 96-well microtiter plates on Day 1, and consecutively evaluated on the LED/image-based MagPix, and laser/flow-based Luminex 200 and FlexMap 3D (all xMAP instruments from Luminex Corp., Austin, TX) on Day 2. The BC assay uses magnetic bead-based fluorescence technology (xMAP, Multi-analyte profiling; Luminex) to simultaneously quantify 10 protein analytes in urine specimens [i.e., angiogenin (ANG), apolipoprotein E (ApoE), carbonic anhydrase IX (CA9), CXCL8/interleukin-8 (IL-8), matrix metalloproteinase-9 (MMP-9), matrix metalloproteinase-10 (MMP-10), serpin A1/alpha-1 anti-trypsin (A1AT), serpin E1/plasminogen activator inhibitor-1 (PAI-1), CD138/syndecan-1 (SDC1), and vascular endothelial growth factor-A (VEGF-A)]. All three analyzers quantify fluorescence signals generated by the Oncuria assay.

**Results:**

All three platforms categorized all 10 analytes in identical samples at nearly identical concentrations, with variance across systems typically < 5%. While the most contemporary instrument, the FlexMap 3D, output higher raw fluorescence values than the two comparator systems, standard curve slopes and analyte concentrations determined in urine samples were concordant across all three units. Forty-four percent of BC samples registered ≥ 1 analyte above the highest standard concentration, i.e., A1AT (n = 7/18), IL-8 (n = 5), and/or ANG (n = 2), while only one control sample registered an analyte (A1AT) above the highest standard concentration.

**Conclusion:**

Multiplex BC assays generate detailed molecular signatures useful for identifying BC, predicting treatment responsiveness, and tracking disease progression and recurrence. The similar performance of the Oncuria assay across three different analyzer systems supports test adaptation by clinical and research laboratories using existing xMAP platforms.

*Trial Registration*: This study was registered at ClinicalTrials.gov as NCT04564781, NCT03193528, NCT03193541, and NCT03193515.

**Supplementary Information:**

The online version contains supplementary material available at 10.1186/s12967-023-04811-2.

## Introduction

Bladder cancer (BC) is the second most common urogenital malignancy, the sixth most common cancer in men (5% of all cancers excluding non-melanoma skin cancer), and the 17th most common cancer in women (1.5% of cases) [1]. Of the 85,000 annual BC diagnoses in the USA [2], ≈75% will be non-muscle-invasive disease (NMIBC) that require years-long monitoring for recurrence and progression after undergoing initial transurethral resection and/or Bacillus Calmette-Guerin (BCG) therapy. Cystoscopy and voided urine cytology remain the gold standards for evaluating BC status [3, 4]. Cystoscopy is uncomfortable, invasive, and carries significant costs and risks (*e.g.*, infection, trauma). Voided urine cytology is noninvasive, economical, and has high specificity for BC but also has suboptimal sensitivity, especially with low-grade and early-stage tumors [5]. Biological marker evaluation in urine samples has evolved as a noninvasive means to more effectively identify BC, stratify patient risk, and monitor treatment progress [6].

Because BC is a heterogeneous disease with varied underlying molecular signatures, no single urine biomarker currently exists that can definitively identify and track disease, or predict the likelihood of recurrence or responsiveness to treatments such as BCG [6–9]. Additionally, the levels of certain individual protein-based markers (e.g., nuclear matrix protein 22, NMP22, and bladder tumor antigen, BTA) are increased in urine in scenarios such as inflammation unrelated to BC [10, 11], which can lead to false-positive interpretations. Evaluating a single BC biomarker in urine samples may be a useful adjunctive test for confirming findings by cystoscopy and histology but remains insufficient for primary diagnosis and treatment planning [3, 4].

Multiplex assays that simultaneously evaluate diverse BC biomarkers in urine increases the likelihood of correctly identifying neoplasms of variable etiology and presentation, predicting treatment response, and accurately tracking therapy effectiveness [5]. These noninvasive approaches generate comprehensive patient-specific BC molecular profiles that can better inform diagnosis and personalized treatment planning, ultimately resulting in improved outcomes [5]. Oncuria^®^ (Nonagen Bioscience Corporation, Los Angeles, CA) is a bead-based multiplex fluorescence immunoassay that coordinately measures 10 protein biomarkers in urine samples [12–15]. Biomarker levels are converted into composite risk scores using differently-weighted algorithms tailored for assisting BC diagnosis, predicting response to BCG therapy in early-stage intermediate to high-risk disease, or tracking treatment progress. The assay is CE marked in Europe and was assigned FDA Breakthrough Device status for expedited review in the USA [16]. The current study compared assay performance and output when urine samples were evaluated with the Oncuria assay using three different fluorescence-analyzing instruments commonly used in diagnostic laboratories worldwide.

## Materials and methods

### Subjects and urine samples

Subjects included 18 individuals bearing BC (17 de novo and 1 recurrent) and 18 non-BC controls (15 with voiding dysfunction/hematuria and 3 with a history of BC on surveillance). Data are reported according to PROBE criteria [17]. Exclusion criteria were a history of renal insufficiency (*i.e.*, glomerular filtration rate < 60 mL/min) and/or reduced urinary creatinine (< 40 mg/dL), because these conditions can cause proteinuria that can interfere with protein immunoassays. Midstream voided urine samples that had been collected for cytology were centrifuged at 1,000 × g for 10 min, with supernatants frozen and undergoing only one freeze–thaw cycle before multiplex analysis. This study received approval and a waiver of consent to use previously banked de-identified urine samples from the Cedars-Sinai Medical Center Institutional Review Board, Los Angeles, CA (IRB #00001459). Study performance complied with the tenets of the Declaration of Helsinki.

### Oncuria assay kit

The Oncuria bead-based fluorescence assay (Nonagen product number DC-03-1001) simultaneously evaluates 10 protein analytes [serpin A1/alpha 1 anti-trypsin (A1AT), angiogenin (ANG), apolipoprotein E (ApoE), carbonic anhydrase IX (CA9), CXCL8/interleukin-8 (IL-8), matrix metalloproteinase-9 (MMP-9), matrix metalloproteinase-10 (MMP-10), serpin E1/plasminogen activator inhibitor-1 (PAI-1), CD138/syndecan-1 (SDC1), and vascular endothelial growth factor-A (VEGF-A)] in voided urine samples, using Luminex xMAP (multiple analyte profiling) technology (Luminex Corp.) [18]. Within a single sample, Oncuria simultaneously captures the 10 analytes using a pool of 10 distinct 6.5-µm magnetic bead + antibody sets, with each bead set differentiated by a unique internal fluorescent label. Beads are recovered, identified, and their captured target antigens quantified on analyzers that measure fluorescence signal intensity. Oncuria is in clinical trials to support FDA approval as an in vitro diagnostic test for predicting BCG response in patients with BC (Oncuria-Predict) [19], for detecting de novo BC in patients with hematuria (Oncuria-Detect) [20, 21], for detecting recurrent BC in patients with a history of BC (Oncuria-Monitor) [22]. In a recent clinical validation study to detect de novo BC, the assay demonstrated an Area Under Receiver Operating Curve, AUROC, value of 0.95 (95% CI 0.90–1.00), with 93% specificity and 93% sensitivity, and PPV of 0.65 and NPV of 0.99 (Table 1) [12]. In a pilot study to predict responsiveness to intravesical BCG therapy for the treatment of NMIBC, the assay demonstrated an AUROC value of 0.89 (95% CI: 0.80–0.99), with a test sensitivity of 82% and a specificity of 85% [13].

**Table 1:** Diagnostic performance of Oncuria assay in identifying high-grade/low-grade and high-stage/low-stage BC

Tumor Grade	Number of BC cases predicted by biomarker assay	AUC	Sensitivity (%)	Specificity (%)	NPV (%)	PPV (%)
Overall	42/45^a^	0.95	0.93	0.93	0.99	0.65
Low-grade tumors^b^	8/9	0.94	0.89	0.93	1.00	0.26
High-grade tumors^b^	34/36	0.95	0.94	0.93	1.00	0.60
NMIBC	25/27	0.93	0.93	0.93	0.99	0.52
MIBC	15/16	0.97	0.94	0.93	1.00	0.39

*NMIBC* non-muscle-invasive bladder cancer, *MIBC* muscle-invasive bladder cancer, *AUC* Area under ROC curve, *NPV* negative predictive value, *PPV* positive predictive value

^a^1 case was missing a single analyte and thus excluded

^b^Per urogenital tumor classification scheme of the World Health Organization, 2022

N = 362 subjects presenting for bladder cancer evaluation. Instrumentation was Luminex 100/200 analyzer

Adopted from Hirasawa et al. [12] in accordance with unrestricted Creative Commons Attribution 4.0 International License BY-4.0

### xMAP instrumentation

The assay was run on the LED/image-based MagPix, and laser/flow-based Luminex 200 and FlexMap 3D xMAP instruments operated with xPONENT Software V4.2 (MagPix and FlexMap 3D) and V4.3 (Luminex 200) (all from Luminex Corp.) [23]. The classic 200 unit is designed for multiplex analysis up to 100 analytes in a single sample, and reads 96-well microtiter plates in ≈ 45 min. The MagPix instrument is more compact and portable than the 200 model to accommodate settings with space constraints or fieldwork, and simultaneously measures 50 analytes in 96-well plates in ≈ 60 min. Both the 200 and MagPix models provide single-digit picogram/mL sensitivity for protein targets and ≥ 3.5 logs of dynamic range. The newer FlexMap 3D allows evaluation of up to 500 analytes in a single sample. It has increased sensitivity (sub-picogram/mL) and dynamic range (≥ 4.5 logs) compared to earlier instruments, and accommodates high-throughput analysis and more advanced automation. The FlexMap 3D reads 96-well plates in ≈ 20 min and 384-well plates in ≈75 min.

### Experimental overview

Voided urine samples were passively thawed at 4 °C and centrifuged at 15,000 × *g* for 10 min at 4 °C to remove potential particulates. Samples, standards, and controls (50 μL/well) were added to a 96-well plate in duplicate wells per condition. Standards comprised a pool of the 10 analytes, from which a seven-point three-fold dilution series was created that covered the dynamic range (> 3-log) of every analyte. On Day 1, the Oncuria assay’s targeted bead set was incubated with sample/standards, followed by decoration of analytes captured by beads using a cocktail of 10 analyte-specific biotinylated primary antibodies followed by washing and incubation with fluorescent phycoerythrin-coupled streptavidin secondary detection reagent. Assays were performed on three 96-well plates (one for each instrument). After assay reaction completion, plates containing sample-reacted and fluorescently-decorated beads were covered with an adhesive aluminum foil seal and stored overnight in the dark at 4 °C, awaiting analysis the next day. Beads targeting individual analytes are distinguishable by unique fluorescent labels incorporated within beads during manufacture. On the morning of Day 2, the FlexMap-delegated plate was warmed to ambient temperature (18‒22 °C). Beads were immobilized by placing the plate on a magnetic separator for 2 min followed by wash buffer aspiration. Beads were then resuspended in 150 µL fresh wash buffer, shaken for 2 min to assure uniform distribution, and then assayed on the FlexMap 3D instrument. At midday and late afternoon of Day 2, the Model 200- and MagPix-designated plates, respectively, had beads immobilized, washed, resuspended, and evaluated on the appropriate instrument, as detailed.

### Data analysis

Data were analyzed using Prism v.9 graphing and statistical analysis software (GraphPad Software, Inc., San Diego, CA), and Excel v.16 (Microsoft Inc., Redmond, WA). Analyte concentrations were determined by comparing sample readings to standard curves generated using a 5-parameter logistical curve fit algorithm (xPONENT software from Luminex). Analyte concentrations are presented as pg/mL ± SD, range, median fluorescence intensity units (MFI, the instruments’ raw data output) or number (% of samples), as appropriate. Mean values were compared by repeated measures ANOVA with Tukey post-test for multiple comparisons. For protein calculations, analyte measurements above the highest standard curve value were replaced with that analyte’s respective highest standard value, as is performed when calculating clinical risk scores.

## Results

### Analyte detection ranges

**Fig. 1:**
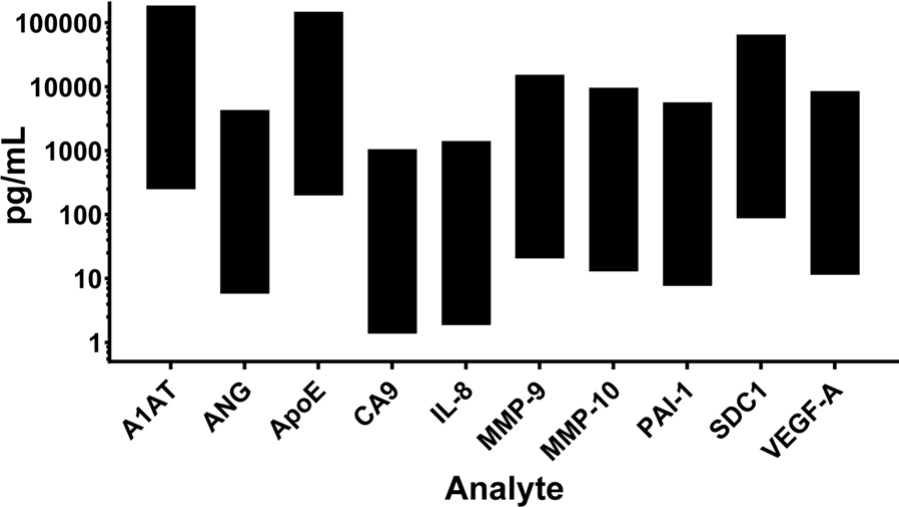
Detection ranges of the 10 bladder cancer biomarkers simultaneously analyzed by the assay

The dynamic range of quantification (lowest to highest standard concentration) for the 10 analytes are shown in Fig. 1. The most sensitive of the 10 concurrently performed assays was for CA9, with a lower detection limit of 1.4 pg/mL. The greatest upper detection limit was for A1AT, at 185,250 pg/mL.

### Subject characteristics

Urine samples were obtained from 18 subjects with a BC diagnosis and 18 control subjects who presented for a voiding condition (Table 2). Most participants were aged ≥ 65 years (53%) and were male (94%). Of the 18 individuals with BC, 50% had Stage T2‒4 disease and 89% had high-grade neoplasms.

**Table 2:** Subject characteristics

Parameter	Controls	Bladder cancer
	N = 18	N = 18
Age, years, mean (range)	53.7 (19‒79)	65.4 (20‒87)
Male:female ratio	18:0	16:2
Race		
White	8	14
Other	6	3
Unknown	4	1
Primary tumor stage		
NMIBC (Ta, Tis, or T1)	N/A^a^	9
MIBC (T2‒T4)	N/A	9
Grade		
Low	N/A	2
High	N/A	16

^a^*N/A* not applicable

### Signal strength by instrument

Raw fluorescence signals output by the Model 200 and MagPix instruments were very similar for all 10 analytes, and both instruments’ outputs were lower than signals from the FlexMap 3D instrument (Table 3). This is due to differences in the optical platforms used in the different instruments, and does not impact analyte concentration determinations.

**Table 3:** Raw fluorescence data outputs across three flow analyzers (median fluorescence intensity, arbitrary units)

Sample ID	Instrument	A1AT	ANG	ApoE	CA9	IL-8	MMP-9	MMP-10	PAI-1	SDC1	VEGF-A
#0003	MagPix	3324	4901	1927	17	5712	1555	240	3062	2202	4163
Tumor	200	3407	4837	2085	20	5646	1694	273	3446	2439	4225
	FlexMap 3D	26,883	38,409	16,223	140	43,608	12,595	2253	26,844	19,223	32,058
#0146	MagPix	2889	1612	514	2	2876	150	3	1412	1663	962
Tumor	200	2991	1604	556	2	3090	185	4	1522	1782	1062
	FlexMap 3D	236,110	13,055	4246	17	23,851	1265	25	11,801	14,059	8080
#0147	MagPix	3825	325	22	0	406	32	0	72	579	20
Tumor	200	3774	342	27	0	467	45	1	81	664	27
	FlexMap 3D	28,626	2564	183	0	3352	262	0	618	4793	181
#0010	MagPix	105	5	6	0	1	1	1	0	131	9
Control	200	125	6	7	2	1	0	1	0	157	14
	FlexMap 3D	868	45	52	0	8	2	6	7	1083	96
#0145	MagPix	1226	22	251	0	6	1	0	2	2007	76
Control	200	1349	27	292	0	7	1	1	1	2257	95
	FlexMap 3D	10,045	176	2138	0	50	2	1	26	16,745	635
#0150	MagPix	128	1	1	0	1	1	1	1	443	0
Control	200	148	2	3	1	1	0	2	0	501	1
	FlexMap 3D	1039	18	5	1	6	0	5	4	3696	5

Shown are raw data outputs from six representative urine samples, from three confirmed BC subjects (“Tumor”) and three Control subjects. Values are averages of duplicate wells per analyte, per subject, rounded to the nearest whole number

### Biomarker quantification by instrument

**Fig. 2:**
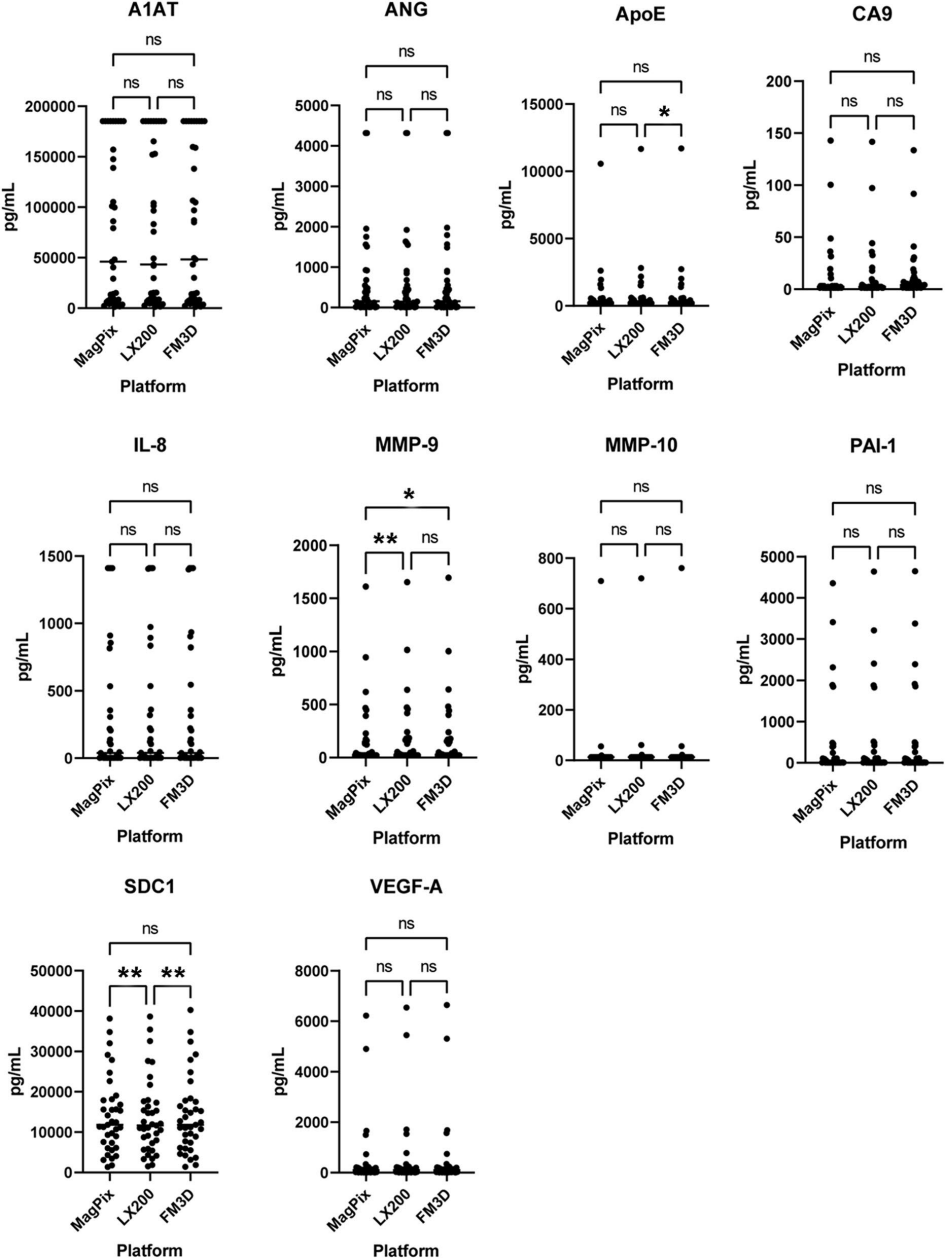
Biomarker protein concentrations across three flow instruments. Calculated protein levels were very similar across platforms, for all 10 analytes. Instrument Abbreviations: LX200 = Luminex 200; FM3D = FlexMap 3D. *p < 0.05; **p < 0.01

The calculated concentration of all 10 analytes was very similar across all three instruments, in 100% (36/36) of urine samples (Table 4, Fig. 2, Additional file 1: Table S1). Although there were statistical discrepancies in mean protein concentrations determined across instruments for three biomarkers (Table 5). For example, MMP-9 concentrations were mathematically different between the MagPix and 200 instruments, but the discrepancy was only ≈ 5%. The four other statistically significant mismatches had even lesser percentages differences between mean analyte concentrations, i.e*.*, 2.2–4.4%.

**Table 4:** Biomarker protein concentrations in urine samples compared across three analyzers (pg/mL)

Sample ID	Instrument	A1AT	ANG	ApoE	CA9	IL-8	MMP-9	MMP-10	PAI-1	SDC1	VEGF-A
#0003	MagPix	185,250	4320	10,566	49	1410	1610	709	4356	38,128	6225
Tumor	200	185,250	4320	11,668	44	1410	1652	720	4639	38,657	6545
	FlexMap 3D	185,250	4320	11,707	41	1410	1694	761	4649	40,278	6646
#0146	MagPix	185,250	1952	2618	11	855	161	13	1889	29,133	1496
Tumor	200	185,250	1922	2823	6	893	179	13	1832	27,662	1535
	FlexMap 3D	185,250	1978	2728	10	903	170	13	1914	29,256	1567
#0147	MagPix	185,250	479	204	1	138	36	13	103	11,336	50
Tumor	200	185,250	475	204	1	140	46	13	109	11,115	57
	FlexMap 3D	185,250	464	204	1	139	40	13	106	11,111	53
#0010	MagPix	3570	28	204	1	2	21	13	8	3099	25
Control	200	3872	25	204	4	2	21	13	8	3306	29
	FlexMap 3D	3581	28	204	1	2	21	13	8	3155	28
#0145	MagPix	48,207	71	1326	1	2	21	13	8	34,823	161
Control	200	49,254	73	1519	1	3	21	13	8	35,446	177
	FlexMap 3D	49,624	71	1408	2	3	21	13	8	34,831	166
#0150	MagPix	4288	12	204	1	2	21	13	8	8971	12
Control	200	4539	11	204	1	2	21	13	8	8725	12
	FlexMap 3D	4247	12	204	4	2	21	13	8	8898	12

Shown is output from six representative urine samples, from three confirmed BC subjects (“Tumor”) and three Control subjects. Values are averages of duplicate wells per analyte, per subject, rounded to the nearest whole number. Values that exceeded the highest standard curve concentration for any individual analyte were assigned that biomarker’s highest standard value. The full dataset for all 36 subjects is provided in Additional file 1: Table S1

**Table 5:** Details of instrument output discrepancies with statistical significance

Biomarker	Instruments	Mean 1 (pg/mL)	Mean 2 (pg/mL)	∆ (pg/mL)	% Difference^a^ (%)	95% CI (pg/mL)	P-value, adjusted^†^
ApoE	200 vs FM3D	819.5	796.1	22.4	2.8	4.6 to 40.1	0.0107
MMP-9	MPX vs 200	166.7	175.1	− 8.4	5.0	− 14.0 to − 2.9	0.0020
	MPX vs FM3D	166.7	174.1	− 7.4	4.4	− 14.0 to − 0.8	0.0261
SDC1	MPX vs 200	13,986.0	13,690.0	295.3	2.2	91.9 to 498.8	0.0031
	200 vs FM3D	13,690.0	13,991.0	− 301.1	2.2	− 494.2 to − 108.1	0.0015

^a^Differences calculated as the absolute value of the ∆ divided by the lower of the two mean protein values × 100, with % differences rounded to nearest 0.1%

^†^P-values calculated by repeated measures ANOVA with Tukey post-test correction for multiple comparisons. All concentration values rounded to nearest 0.1 picogram

Instrument Abbreviations: 200 = Luminex 200; FM3D = FlexMap 3D; MPX = MagPix

While this report is intended to demonstrate assay reproducibility across different xMAP instruments and not for clinical validation, noteworthy elevations of analytes were not noted in BC versus Control urine samples (Table 4).

### Values exceeding dynamic range

All three instruments captured and defined all 10 biomarkers at or below their highest analyte-specific standard curve concentration in nearly all urine samples (Additional file 1: Table S1). Of the BC samples, 44% (8/18) registered at least one analyte above the highest standard curve concentration, observed with A1AT (n = 7/18), IL-8 (n = 5) and/or ANG (n = 2); in Control samples, a single instance was observed of a biomarker (i.e., A1AT) exceeding the assay’s dynamic range. In 11/15 instances, the dynamic range was exceeded with all 3 instruments (Additional file 1: Table S1).

The slopes of standard curves generated by all three instruments were essentially identical at all points in the assay range, for all analytes (not shown).

## Discussion

This study confirmed reproducible assay performance when voided urine samples were interrogated by the Oncuria multiplex BC assay, with very similar data output obtained from three different xMAP analyzers commonly used in diagnostic and research laboratories. Clinical validation studies of the Oncuria assay have demonstrated its ability to accurately discriminate BC patients from healthy controls, and its potential for identifying BC, predicting therapeutic responsiveness, tracking treatment progress, and monitoring for recurrence [12–15]. The current demonstration that the concentrations of all 10 BC biomarkers were adjudicated nearly identically across three instrument platforms indicates that the Oncuria assay is highly amenable to standardization across laboratories that use different xMAP systems [24].

Multiplex assays that evaluate a composite molecular signature in urine have greater utility in detecting and monitoring BC than efforts to identify a single BC biomarker [6, 7, 10, 11]. Advantages of multiplex immunoassays include increased efficiency and lower costs versus evaluating multiple analytes individually, and high-throughput capabilities that are further enhanced by using the automated features of modern instrumentation platforms [25]. The practical utility of generating unique biomarker signatures is highlighted by the recent increase in FDA approvals of multiplex proteomic assays for clinical use, including cancer detection [25, 26]. The molecular profile of the 10 biomarkers is converted into a BC risk score based on the relative contribution of individual analytes; ongoing research goals include adjusting and optimizing the Oncuria assay’s algorithm based on patient demographics and medical history to provide more opportunities for personalized application [27].

Cystoscopy and voided urine cytology remain the frontline methods for assessing BC status [3, 4]. Urine testing is a noninvasive approach without the safety risks of cystoscopy, which becomes particularly important in elderly and frail patients. Cystoscopy is sensitive for papillary lesions but tends to miss flat lesions such as carcinoma in situ (CIS), although newer imaging techniques provide improved contrast to differentiate tumor from normal tissue [28]. While cystoscopy is often used in individuals with NMIBC and in MIBC patients who have undergone bladder-sparing treatments, no global consensus exists for endoscopic follow-up scheduling [29]. There is growing evidence that cystoscopy may be overutilized, increasing both direct treatment costs and risks [30]. In NMIBC patients, cystoscopy overuse has been linked to a twofold increase in transurethral resections performed, with an increased proportion of resection specimens not containing cancer, thus attesting to the difficulty in visually identifying cancers [31]. While urine cytology is noninvasive, it often produces indeterminate (atypical) diagnosis and has suboptimal sensitivity for detecting early and low-grade tumors [5]. One prospective study reported cytology sensitivities of 84% for high-grade but only 16% for low-grade NMIBC [32]. By contrast to cystoscopy and cytology, the Oncuria assay, when adjusted for patient demographics, previously showed sensitivity values for high-grade BC, low-grade BC, MIBC and NMIBC of 94%, 89%, 97% and 93%, respectively (using the Model 200 flow analyzer) [12]. The 10-analyte panel also had a negative-predictive value of 99% for a BC diagnosis, which may prevent superfluous testing and procedures. Oncuria testing may be an important noninvasive adjunctive method for confirming and adding clinical value to the BC findings of cystoscopy and cytology.

A primary study limitation was that the three xMAP instruments compared were produced by one manufacturer. Fluorescent bead-based assays are easily standardized to ensure inter-lab reproducibility [24, 33, 34]. Generalization of findings is limited by the inclusion of primarily male urine samples, though prior and ongoing studies have included a larger number of samples from females. Additionally, updated algorithms used to analyze BC risk with the Oncuria assay take gender into account for clinical interpretation of assay output [12]. While MIBC accounts for ≈ 25% of BC cases, our study employed a higher percentage (50%) due to our hospital being a tertiary institution that sees many advanced BC cases. While this discrepancy has relevance in a clinical evaluation, it has less bearing on the intent or outcome of the current methodological investigation.

In conclusion, the Oncuria BC assay performed similarly well across three different analysis platforms for all 10 analytes simultaneously evaluated in urine samples. This agreement across instruments indicates that the test is amenable to standardized performance in laboratories using existing xMAP, without requiring costly outlays for new equipment. The multiplex Oncuria assay shows promise as a noninvasive and rapid-reporting adjunctive approach to cystoscopy and cytology in helping to identify BC, predict disease response to various therapies, track treatment progress, and monitor for recurrence.

### Supplementary Information

**Additional file 1:** **Table S1. **Concentrations of all 10 analytes in all 36 urine samples.

## Data Availability

The anonymized datasets used and/or analyzed during the current study are available from the corresponding author upon reasonable request.
