# Supplementary material for: Bladder cancer risk stratification with the Oncuria 10-plex bead-based urinalysis assay using three different Luminex xMAP instrumentation platforms
Source: J Transl Med. 2024 Jan 2;22:8. doi: 10.1186/s12967-023-04811-2 (PMC10763405; doi:10.1186/s12967-023-04811-2)
Supplement: Supplementary file 1 — Additional file 1: Table S1. Concentrations of all 10 analytes in all 36 urine samples. [file 12967_2023_4811_MOESM1_ESM.pdf]

Additional file. Table S1. Concentrations of all 10 analytes in all 36 urine samples.

		Concentration (pg/mL); bolding indicates analyte outside dynamic range									
**Sample ID**	**Instrument**	**MMP-9**	**IL-8**	**VEGF**	**CA9**	**SDC1**	**PAI1**	**ApoE**	**A1AT**	**Angiogenin**	**MMP-10**
Cancer-1	**MagPix**	1610	**1852**	6225	49	38128	4356	10566	**1830163**	**7244**	709
	**LX200**	1652	**1871**	6545	44	38657	4639	11668	**1088096**	**7938**	720
	**FlexMap 3D**	1694	**1830**	6646	41	40278	4649	11707	**2069855**	**7734**	761
Control-1	**MagPix**	**11**	2	25	1	3099	8	204	3570	28	13
	**LX200**	21	**0**	29	4	3306	8	204	3872	25	**8**
	**FlexMap 3D**	**2**	**1**	28	1	3155	**2**	204	3581	28	**4**
Cancer-2	**MagPix**	24	38	**9**	2	12619	9	**72**	9679	124	13
	**LX200**	27	39	**9**	1	11935	**3**	**106**	10117	124	13
	**FlexMap 3D**	29	38	**9**	3	12740	11	**62**	10033	127	**3**
Control-2	**MagPix**	**0**	2	161	1	34823	**3**	1326	48207	71	13
	**LX200**	21	3	177	1	35446	8	1519	49254	73	13
	**FlexMap 3D**	**2**	3	166	2	34831	**6**	1408	49624	71	**2**
Cancer-3	**MagPix**	161	855	1496	11	29133	1889	2618	356744	1952	**4**
	**LX200**	179	893	1535	6	27662	1832	2823	518640	1922	**10**
	**FlexMap 3D**	170	903	1567	10	29256	1914	2728	424380	1978	**11**
Cancer-4	**MagPix**	36	138	50	1	11336	103	**99**	185250	479	13
	**LX200**	46	140	57	1	11115	109	**68**	185250	475	**8**
	**FlexMap 3D**	40	139	53	**1**	11111	106	**92**	185250	464	**2**
Control-3	**MagPix**	50	2	14	2	1335	**5**	204	7924	80	13
	**LX200**	58	3	16	2	1521	10	204	8458	83	**4**
	**FlexMap 3D**	56	3	16	6	1390	9	204	8160	87	**4**
Control-4	**MagPix**	**11**	2	12	1	8971	**5**	204	4288	12	13
	**LX200**	21	**1**	12	1	8725	8	204	4539	11	**4**
	**FlexMap 3D**	**1**	**1**	12	4	8898	**2**	204	4247	12	**4**
Control-5	**MagPix**	21	2	**6**	1	10722	8	**104**	4733	8	13
	**LX200**	21	**0**	12	1	10525	8	**66**	5129	6	**7**
	**FlexMap 3D**	**2**	2	12	2	10761	**2**	**84**	4981	10	**4**

Cancer-5	**MagPix**	468	119	65	3	7353	87	414	86049	549	**3**
	**LX200**	472	121	69	1	7274	89	435	83292	548	**6**
	**FlexMap 3D**	481	121	67	4	7377	88	418	87133	550	**7**
Cancer-6	**MagPix**	395	356	194	31	16813	404	1939	**232308**	185	**3**
	**LX200**	418	360	202	33	16369	422	2185	**282169**	189	**8**
	**FlexMap 3D**	402	358	198	29	16432	416	2024	**216989**	186	**7**
Control-6	**MagPix**	42	11	733	3	12438	3	309	99831	156	**8**
	**LX200**	52	11	773	1	12094	8	322	96606	149	**7**
	**FlexMap 3D**	49	12	746	5	12446	6	309	96885	155	**4**
Cancer-7	**MagPix**	140	2944	190	19	18133	487	482	147543	1561	13
	**LX200**	157	3245	197	18	17645	513	513	165189	1600	**7**
	**FlexMap 3D**	150	2805	198	16	17813	497	471	158844	1565	**2**
Cancer-8	**MagPix**	946	909	149	11	11792	388	539	292457	466	13
	**LX200**	1014	973	168	7	11627	406	580	449407	448	**2**
	**FlexMap 3D**	1003	935	166	10	11796	393	554	291296	449	**2**
Cancer-9	**MagPix**	619	2410	335	36	15597	2320	343	692609	929	20
	**LX200**	640	2451	337	36	15292	2410	358	185250	911	22
	**FlexMap 3D**	643	2229	344	31	15642	2392	336	850853	911	22
Control-7	**MagPix**	**3**	**1**	25	1	19063	**4**	586	6133	11	13
	**LX200**	**12**	**2**	28	1	17936	8	607	6564	13	13
	**FlexMap 3D**	**6**	**2**	25	3	18335	**5**	588	6238	**3**	**3**
Cancer-10	**MagPix**	**20**	51	119	3	15634	9	380	138839	23	13
	**LX200**	21	52	127	2	15386	**5**	408	152941	17	**4**
	**FlexMap 3D**	26	51	128	4	15228	11	381	138154	21	**5**
Control-8	**MagPix**	173	103	226	3	11134	23	1336	**202430**	1511	56
	**LX200**	184	104	234	2	10824	22	1503	**232297**	1552	61
	**FlexMap 3D**	177	105	235	7	11061	23	1384	**204275**	1486	56
Control-9	**MagPix**	**11**	2	12	1	15514	8	**76**	15041	13	13
	**LX200**	21	**1**	12	2	14718	8	204	15402	13	13
	**FlexMap 3D**	**2**	**0**	12	2	15378	**2**	**67**	15005	13	**2**

Control-10	**MagPix**	**0**	2	32	1	4327	8	**17**	9284	13	13
	**LX200**	21	**1**	37	2	4339	8	204	9804	16	**10**
	**FlexMap 3D**	**4**	2	32	4	4592	**3**	**10**	9708	11	**3**
Cancer-11	**MagPix**	**1**	306	53	3	3443	58	**183**	79277	60	13
	**LX200**	21	319	60	2	3380	61	**170**	75669	55	**9**
	**FlexMap 3D**	**4**	314	61	4	3631	60	**194**	85005	64	**2**
Cancer-12	**MagPix**	**8**	9	135	1	5531	**5**	**41**	8411	380	13
	**LX200**	**8**	9	137	2	5447	8	204	8518	379	**4**
	**FlexMap 3D**	**11**	9	139	2	5536	**2**	**32**	8291	379	**6**
Cancer-13	**MagPix**	**0**	4	**6**	1	7561	**3**	204	13479	78	13
	**LX200**	**11**	5	**7**	1	7990	8	204	15300	85	**7**
	**FlexMap 3D**	**4**	5	**4**	2	7712	**6**	204	13883	82	**2**
Cancer-14	**MagPix**	30	219	13	2	6019	105	204	101080	93	13
	**LX200**	36	222	12	2	5853	111	204	101420	93	**8**
	**FlexMap 3D**	35	222	14	5	6101	104	204	104773	95	**3**
Cancer-15	**MagPix**	142	**8915**	4902	100	14130	3412	411	**1371051**	1754	13
	**LX200**	166	1410	5452	97	12568	3212	425	185250	1636	**2**
	**FlexMap 3D**	154	**15947**	5311	92	13746	3378	413	**3131129**	1793	**2**
Cancer-16	**MagPix**	13	205	211	143	9738	243	254	29319	680	13
	**LX200**	12	209	216	142	9699	268	276	29821	683	**8**
	**FlexMap 3D**	15	206	212	134	9643	260	260	30144	668	**2**
Cancer-17	**MagPix**	450	816	79	19	1774	108	204	157020	220	13
	**LX200**	457	834	86	21	1873	112	204	152076	216	**7**
	**FlexMap 3D**	443	822	80	19	1873	109	204	159708	218	**2**
Cancer-18	**MagPix**	122	1439	1658	14	17900	1846	1599	**276990**	**4353**	13
	**LX200**	131	1406	1712	10	17296	1881	1753	**423433**	**4790**	**9**
	**FlexMap 3D**	131	1401	1686	12	17537	1858	1620	**307073**	**4372**	**1**
Control-11	**MagPix**	**0**	2	123	1	9301	**1**	**65**	7320	228	**4**
	**LX200**	21	**1**	130	3	9102	8	**114**	7882	228	**11**
	**FlexMap 3D**	**3**	2	135	1	9338	**1**	**68**	7462	227	**10**

Control-12	**MagPix**	228	9	150	2	11263	**3**	**74**	40481	404	13
	**LX200**	241	10	153	2	11006	8	**25**	42023	408	**8**
	**FlexMap 3D**	239	10	160	5	11239	**5**	**66**	43225	408	**2**
Control-13	**MagPix**	**11**	2	**9**	1	4070	8	204	1670	21	13
	**LX200**	21	**1**	**8**	3	4144	8	204	1659	21	13
	**FlexMap 3D**	**3**	**1**	**8**	**1**	4210	**3**	204	1632	19	**1**
Control-14	**MagPix**	21	2	12	1	22661	8	204	1196	8	13
	**LX200**	21	**1**	12	1	21732	8	204	1211	10	**2**
	**FlexMap 3D**	**2**	2	12	2	22601	**1**	204	1236	**3**	**2**
Control-15	**MagPix**	**11**	**1**	12	1	32041	8	**65**	4209	11	13
	**LX200**	21	**2**	12	1	32568	8	204	4561	**4**	13
	**FlexMap 3D**	**1**	**2**	12	1	32449	**2**	**55**	4195	6	**1**
Control-16	**MagPix**	**5**	44	101	3	6098	23	204	46032	918	**3**
	**LX200**	**4**	44	101	2	5652	22	204	43306	849	**6**
	**FlexMap 3D**	**9**	45	105	8	5956	26	204	48221	882	**8**
Control-17	**MagPix**	**5**	2	50	1	27903	8	204	2272	8	13
	**LX200**	**13**	**1**	55	1	27375	8	204	2503	7	**8**
	**FlexMap 3D**	**7**	**1**	51	1	27948	**1**	204	2306	6	**4**
Control-18	**MagPix**	**3**	4	202	2	24746	**2**	**41**	14070	131	13
	**LX200**	**12**	4	214	2	23695	8	204	14622	125	**8**
	**FlexMap 3D**	**5**	4	211	**1**	24846	**2**	**20**	14303	131	**5**
	Standard1	15320	1410	8500	1050	64960	5670	148740	185250	4320	9600
	Standard7	21	2	12	1	89	8	204	254	6	13
